# PCSK9 Promotes Platelet Activation and NET Formation, Aggravating Pulmonary Microthrombosis in Sepsis-Induced Lung Injury

**DOI:** 10.3390/biomedicines13122843

**Published:** 2025-11-21

**Authors:** Xin Lv, Wanxia Xiong, Linghui Jiang

**Affiliations:** Department of Anesthesiology, Zhongshan Hospital, Fudan University, Shanghai 200032, China; lvxinseven@163.com

**Keywords:** sepsis, pulmonary microthrombosis, PCSK9, platelet activation, neutrophil extracellular traps

## Abstract

**Background**: Sepsis-induced lung injury is a major clinical challenge, frequently complicated by pulmonary microthrombosis, which exacerbates disease severity and worsens prognosis. Recent studies suggest that proprotein convertase subtilisin/kexin type 9 (PCSK9) is involved in inflammatory and thrombotic processes. However, the contribution of PCSK9 to pulmonary microthrombosis in sepsis remains unclear. **Methods**: Adult male C57BL/6J mice were subjected to cecal ligation and puncture (CLP) to induce sepsis. Twelve hours post-CLP, mice received either recombinant PCSK9 (1 μg, i.p.) or the PCSK9 inhibitor evolocumab (10 mg/kg, i.p.). Platelet activation and pulmonary microthrombosis were assessed using hematoxylin–eosin staining, immunofluorescence, and thromboelastography. **Results**: Administration of PCSK9 significantly enhanced platelet activation and increased neutrophil extracellular trap (NET) formation, promoting pulmonary microthrombosis in septic mice. In contrast, administration of evolocumab effectively attenuated platelet activation, reduced NET formation, and alleviated pulmonary microthrombosis. **Conclusions**: PCSK9 exacerbates sepsis-induced pulmonary microthrombosis by promoting platelet activation and NET formation. Targeting PCSK9 may represent a novel therapeutic strategy for preventing thrombotic complications in sepsis-induced lung injury.

## 1. Introduction

Sepsis is a prevalent and frequently life-threatening medical emergency characterized by a high mortality rate and significant long-term morbidity among survivors [1]. Organ dysfunction represents the core pathophysiological mechanism of sepsis and is a major contributor to its high mortality rate. Among the various forms of organ damage associated with sepsis, lung injury constitutes the most prevalent manifestation [2]. It is widely recognized that inflammation and oxidative stress are pivotal contributors to sepsis-induced lung injury [3]. Infection-induced inflammatory responses activate host immune cells, resulting in the release of multiple inflammatory mediators, including cytokines and chemokines [4]. In addition, activation of immune cells can stimulate the coagulation system, promoting the release of platelets and coagulation factors, which leads to thrombus formation and further amplifies the inflammatory response, ultimately resulting in organ dysfunction and death [5,6]. However, the exact mechanism remains unclear. Therefore, further investigation into the underlying pathogenesis is essential to develop effective treatments aimed at reducing the incidence of sepsis-induced pulmonary microthrombosis.

Platelets are the most abundant cellular component of blood after erythrocytes [7]. Initial research on platelets primarily focused on their hemostasis. However, more recent studies have uncovered its key players in inflammation and immune regulation, particularly in sepsis [8]. Both platelets and coagulation are involved in thrombosis, which is generally regarded as a pathological deviation in hemostasis. However, recent findings suggest that intravascular thrombosis also involves processes that are distinct from hemostasis and which occur mainly in pathological situations such as sepsis [9]. The participation of neutrophils and monocytes, as well as dendritic cells leads to a “thrombosis-related signature” which initiates and propagates fibrin formation and triggers platelet activation during the development of thrombosis [10]. Platelets play a central role in thrombotic diseases [11]. Platelet activation contributes to microvascular thrombosis and organ failure in systemic inflammation, particularly in septic conditions [12]. Thus, inhibiting platelet activation is a promising therapeutic strategy, but the upstream regulators of platelet activation in sepsis are not fully understood.

Proprotein convertase subtilisin/kexin type 9 (PCSK9), a serine protease mainly produced in the liver, is well known for its role in LDL cholesterol metabolism [13]. Increasing evidence indicates that PCSK9 exerts a wide range of pleiotropic effects. Recent studies have revealed that PCSK9 also promotes inflammation and thrombosis, independent of its lipid-lowering effects [13,14]. Clinical studies have suggested that PCSK9 plays a crucial role in the pathogenesis of sepsis, and that inhibition of its activity can effectively improve the prognosis of affected patients [15,16]. In a study of patients with recent acute coronary syndrome (ACS) undergoing PCI, serum PCSK9 levels were independently associated with high on-treatment platelet reactivity and an increased incidence of atherothrombotic events [17]. Studies have shown that PCSK9 can enhance the inflammatory response by regulating the TLR4/NF-κB signaling pathway in innate immune cells, which is closely related to the promotion of atherosclerotic inflammation [18]. However, its role in sepsis-induced pulmonary microthrombosis is not well defined.

Platelets promote the response of innate immune cells. Neutrophils and monocytes are the first line of defense of the innate immune system against infection. Activated platelets drive the response of target white blood cells and regulate the host’s response to infection [12]. Neutrophil extracellular traps (NETs) are web-like extrusions of genetic material, which are released upon neutrophil activation [19,20]. NETs have traditionally been implicated in severe infections, such as sepsis, where the release of chromatin, decorated with neutrophil granular proteins, acts as an additional defense mechanism of the innate immune system against circulating pathogens [21]. Studies have shown that neutrophils and platelets play essential synergistic roles in the process of thromboinflammation [22]. Clark et al. demonstrated that platelet toll-like receptor 4 (TLR4) detected TLR4 ligands in blood and induced platelet binding to adherent neutrophils [23]. These interactions lead to robust neutrophil activation and the formation of NETs. NETs ensnare bacteria within the vasculature, primarily in pulmonary capillaries and liver sinusoids. These NETs have a proteolytic activity that can trap and kill microbes in tissues [12]. NETs are an important product of the platelet-neutrophil axis and have been identified as key mediators of the coagulation state in thrombotic diseases [20]. However, the role of PCSK9 in the interaction between platelets and NETs has not yet been fully elucidated. Therefore, this study aims to investigate the increased expression of PCSK9 in a sepsis mouse model, its subsequent activation of platelets, which in turn triggers NET formation, ultimately leading to the development of sepsis-induced pulmonary microthrombosis.

## 2. Materials and Methods

### 2.1. Animals and Sepsis Model

Male C57BL/6J mice (6–8 weeks old, 25–30 g) were purchased from Beijing Vitanova Experimental Animal Technology Co., Ltd., Beijing, China. The mice were housed under standard conditions with free access to food and water. Sepsis was induced by cecal ligation and puncture (CLP) under anesthesia. The cecum was ligated 5 mm from the tip, punctured with a 22-gauge needle, and a small amount of feces was extruded. Sham-operated mice underwent the same procedure without ligation or puncture. Mice were resuscitated with subcutaneous saline and monitored until recovery. All animal procedures were approved by the Animal Ethics Committee of Zhongshan Hospital Fudan University passed the ethical review on 28 February 2022 (No. 2022ZSQN22).

### 2.2. Experimental Design and Drug Administration

This study was completed in two separate experiments. In experiment 1, mice were randomly divided into the control group (*n* = 12) and the sepsis group (*n* = 12). In experiment 2, mice were randomly divided into four groups: control + vehicle group (saline + vehicle), sepsis + vehicle group (CLP + vehicle), sepsis + PCSK9 group (CLP + PCSK9) and sepsis + evolocumab group (CLP + evolocumab); each group consisted of 20 mice. Mice in the sepsis + PCSK9 group were injected with 1 μg of recombinant human PCSK9 (C38M, novoproteen) via the tail vein to increase the expression of PCSK9 in the body [24]. Existing experiment has demonstrated that the PCSK9 inhibitor evolocumab can improve the thrombosis caused by PCSK9 [25]. In our experiment, the mice in sepsis + evolocumab group were injected in tail vein with PCSK9 inhibitor evolocumab (Repatha, Amgen, Thousand Oaks, CA, USA), a single dose of 10 mg/kg.

### 2.3. Histopathology and Immunofluorescence

Lung tissues were fixed, paraffin-embedded, sectioned, and stained with hematoxylin–eosin (HE). For immunofluorescence, tissues were fixed, dehydrated, embedded in OCT, and sectioned. Sections were incubated with primary antibodies LY6G (127613, Biolegend, San Diego, CA, USA), Cit-H3 (97272S, cellsignal, Danvers, MA, USA), CD41 (ab181582, abcam, Cambridge, UK), followed by secondary antibodies and DAPI (1:1000; Beyotime Biotechnology, Shanghai, China). Images were acquired with a confocal microscope and analyzed using ImageJ software (version 1.53).

### 2.4. ELISA

The concentrations of myeloperoxidase (MPO) and platelet factor 4 (PF4) in mouse plasma were detected by using enzyme-linked immunosorbent assay (ELISA) kit (ER0267, XL-ER0689, Xinle Biotechnology Co., Ltd., Shanghai, China). Add the plasma to the enzyme-labeled Wells pre-coated with antibodies, then add the biotin-labeled recognition antigen. Incubate at 37 °C for 30 min. The two compete with the solid-phase antibody for binding to form an immune complex. Wash off the unbound biotin antigen with PBST, and then add avidin-HRP. After incubation at 37 °C for 30 min, avidin-HRP binds to the biotin antigen. After washing, the bound HRP catalyzes tetramethylbenzidine to turn blue, and then converts to yellow under the action of acid. There is an absorption peak at a wavelength of 450 nm, and the absorbance value is negatively correlated with the concentration of the antigen in the sample.

### 2.5. Thromboelastography

The abdominal cavity was accessed through a median incision, and the caudal vena cava was exposed and punctured. Venipuncture was carried out using a 2 mL syringe (25 gauge needle) containing 0.1 mL sodium citrate. A total of 1 mL of citrated whole blood (blood:citrate ratio 10:1) was collected. Subsequently, 340 μL of citrated blood was mixed with 20 μL of CaCl_2_, and thromboelastography (TEG) analysis was immediately initiated using a Hemostasis Analyzer (Haemoscope TEG-5000, Haemonetics Corp., Braintree, MA, USA). The evaluated parameters included R-time (in minutes), indicating the latency until initial fibrin formation, and maximum amplitude (MA, in millimeters), which reflects the ultimate strength of the fibrin clot.

### 2.6. Statistical Analysis

Statistical analyses were conducted using GraphPad Prism 9.0 software. The normality of data distribution was assessed using the Shapiro–Wilk test. Data are presented as mean ± standard deviation (SD), accompanied by dot plots. Comparisons between groups were performed using a two-tailed Student’s *t*-test or one-way analysis of variance (ANOVA) with Tukey’s post hoc test, as appropriate. A value of *p* < 0.05 was considered statistically significant.

## 3. Results

### 3.1. PCSK9 Enhances Platelet Aggregation in Sepsis Mice

**Figure 1:**
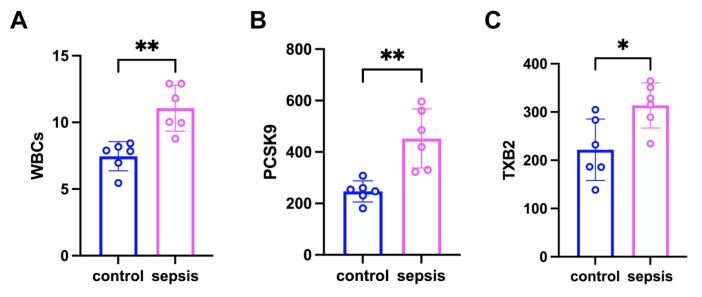
The expression of PCSK9 in the blood of septic mice was increased, and platelet aggregation occurred. (**A**) The expression of WBCs in the blood of the two groups. (**B**) The expression of PCSK9 in the blood of the two groups. (**C**) The expression of TXB2 in the blood of the two groups. Data are shown as the mean ± SD (*n* = 6 mice/group). * *p* < 0.05, ** *p* < 0.01 vs. the indicated groups.

The results of our study demonstrated a significantly elevated expression of white blood cells (WBCs) in the blood of sepsis-induced mice compared to the control group (Figure 1A), thereby confirming the successful establishment of the sepsis model. We observed a significant increase in PCSK9 in the sepsis group compared to the control group (Figure 1B). In addition, in comparison with the control group, the level of TXB2 was significantly increased in the sepsis group (Figure 1C). These results are consistent with the trend of previous experimental results [15,26,27]. These findings indicate that sepsis group exhibited platelet aggregation and were in a prothrombotic state.

**Figure 2:**
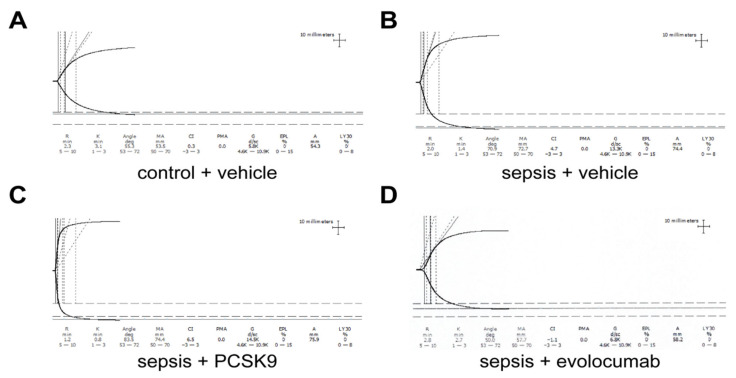
Platelet aggregation enhanced in mice with sepsis. (**A**) The platelet activation of mice in control + vehicle group was detected by TEG. (**B**) The platelet activation of mice in sepsis + vehicle group was detected by TEG. (**C**) The platelet activation of mice in sepsis + PCSK9 group was detected by TEG. (**D**) The platelet activation of mice in sepsis + evolocumab group was detected by TEG. Horizontal axis (X-axis): Time (minutes, min), indicating the time elapsed during the detection process; The vertical axis (Y-axis): amplitude (mm), indicating the strength of the blood clot. Main parameters and clinical significance of TEG: R (Reaction time): The time from the start of detection to the formation of initial fibrin (the curve begins to deviate from the baseline). Reflect the activity of coagulation factors; K (Kinetics time): The period from when the curve starts to deviate from the baseline to when the amplitude reaches 20 mm. Reflect the formation rate of fibrin and the content of fibrin; Alpha angle: The slope Angle of the curve’s ascent, indicating the rate of fibrin polymerization; MA (Maximum Amplitude): The amplitude at which the curve reaches its maximum width, reflecting the maximum strength of the clot (mainly determined by platelet function and fibrin). LY30 (Lysis 30): The percentage decrease in amplitude 30 min after clot formation, indicating fibrinolytic activity.

Next, we used TEG to detect platelet activation in four groups of mice. Our results demonstrated that compared to the control group (Figure 2A), the sepsis + vehicle group exhibited a hypercoagulable state, characterized by a decreased Reaction time (R) value, shortened Kinetics time (K) time, increased Angle, elevated Maximum Amplitude (MA), and enhanced platelet aggregation (Figure 2B). PCSK9 can exacerbate the hypercoagulable state in sepsis, as evidenced by a decreased R value, shortened K time, increased Angle, and elevated MA (Figure 2C). Evolocumab can mitigate the hypercoagulability in sepsis, as indicated by an increased R value, prolonged K time, decreased Angle, and reduced MA (Figure 2D). In summary, sepsis mice exhibited enhanced platelet aggregation compared to control mice. PCSK9 exacerbated this effect, while PCSK9 inhibitor evolocumab attenuated platelet aggregation.

### 3.2. PCSK9 Accelerates Pulmonary Infiltration and Microthrombosis Formation

**Figure 3:**
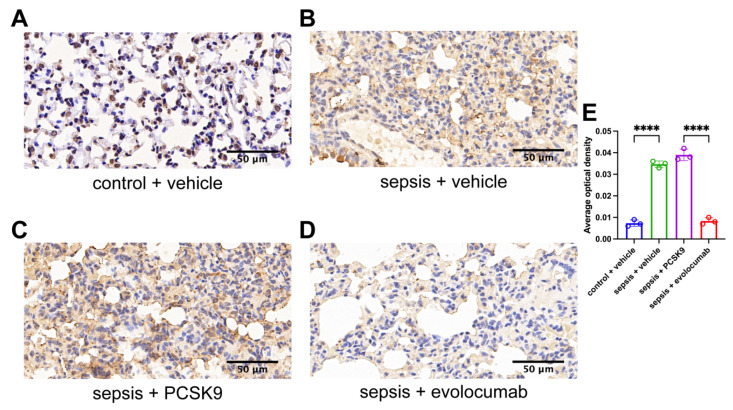
The expression of PCSK9 in the lung tissue of septic mice has increased. (**A**) Histopathology representative image of PCSK9 in lung tissue of mice in control + vehicle group, scale = 50 μm. (**B**) Histopathology representative image of PCSK9 in lung tissue of mice in sepsis + vehicle group, scale = 50 μm. (**C**) Histopathology representative image of PCSK9 in lung tissue of mice in sepsis + PCSK9 group, scale = 50 μm. (**D**) Histopathology representative image of PCSK9 in lung tissue of mice in sepsis + evolocumab group, scale = 50 μm. (**E**) Quantitative analysis of the average optical density value of PCSK9 of lung tissues among four groups. Data are shown as the mean ± SD (*n* = 3 mice/group). **** *p* < 0.0001 vs. the indicated groups.

Compared to the mice in the control + vehicle group, the expression of PCSK9 in the lung tissue of mice in the sepsis + vehicle group showed a modest increase. The same situation can also be detected in the sepsis + PCSK9 group. However, treatment with the PCSK9 inhibitor evolocumab led to a significant reduction in the expression of PCSK9 in the lung tissue of mice with sepsis (Figure 3).

**Figure 4:**
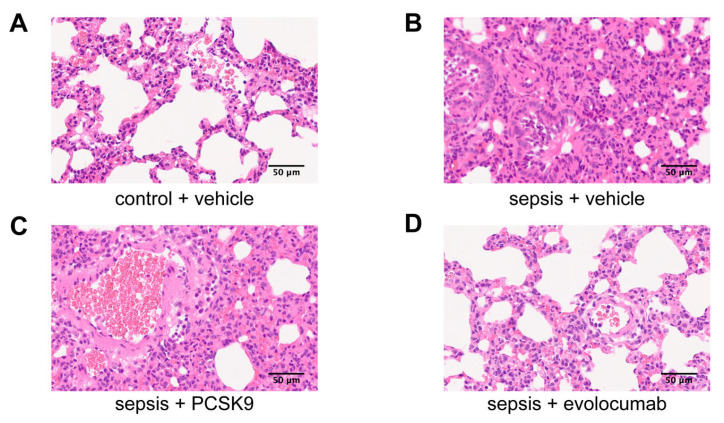
PCSK9 promoted alveolar inflammatory infiltration and fibrinin-like necrosis of the vascular wall in septic mice. (**A**) HE staining representative image of lung tissues in control + vehicle group, scale = 50 μm. (**B**) HE staining representative image of lung tissues in sepsis + vehicle group, scale = 50 μm. (**C**) HE staining representative image of lung tissues in sepsis + PCSK9 group, scale = 50 μm. (**D**) HE staining representative image of lung tissues in sepsis + evolocumab group, scale = 50 μm.

In our experiment, HE staining was used to observe the pathological changes and thrombosis in the lung tissues of the four groups of mice. The results showed that compared with normal lung tissue in control + vehicle group (Figure 4A), the alveolar structure was destroyed, the alveolar wall was significantly thickened, and the blood vessels were dilated and congested. Extensive alveolar and interstitial edema with extensive inflammatory cell infiltration. The epithelial cells of the bronchus were exfoliated, and neutrophil infiltration was observed in the lumen. Pulmonary arteriole edema, epithelial cell shedding, wall fibrinoid necrosis, vascular lumen and periwall neutrophil infiltration in sepsis + vehicle mice (Figure 4B). This condition was also observed in the sepsis + PCSK9 group of mice (Figure 4C). However, the PCSK9 inhibitor evolocumab can improve the situation (Figure 4D). These results suggest that PCSK9 contributes to the promotion of alveolar inflammatory infiltration and fibrinoid necrosis of the blood vessel walls in sepsis mice. However, PCSK9 inhibitor evolocumab has the potential to ameliorate these pathological conditions.

### 3.3. Platelet Activation-Induced NET Formation Drives Pulmonary Microthrombosis

**Figure 5:**
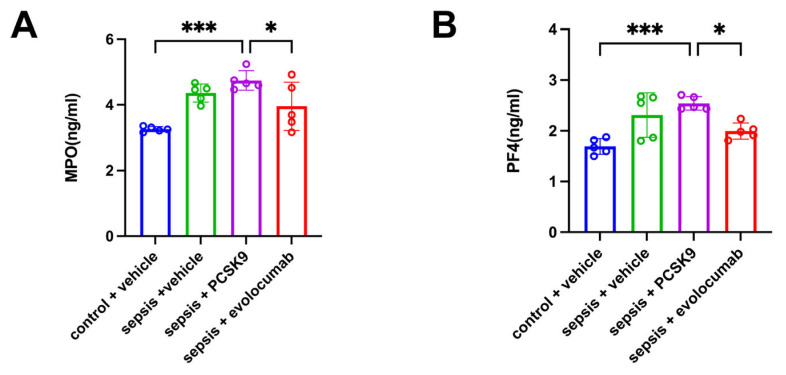
NETs were formed after platelet activation in septic mice. (**A**) ELISA analysis of MPO expression in plasma among the four groups. (**B**) ELISA analysis of PF4 expression in plasma among the four groups. Data are shown as the mean ± SD (*n* = 5 mice/group). * *p* < 0.05, *** *p* < 0.001 vs. the indicated groups.

MPO of plasma was used to detect the level of NETs, and PF4 of plasma was used to reflect the activation status of platelets. Compared to the control + vehicle group, the levels of platelet activation and NETs were elevated in the sepsis + vehicle group. PCSK9 exacerbated platelet activation and NETs levels in sepsis mice, while PCSK9 inhibitor evolocumab reduced these levels (Figure 5).

**Figure 6:**
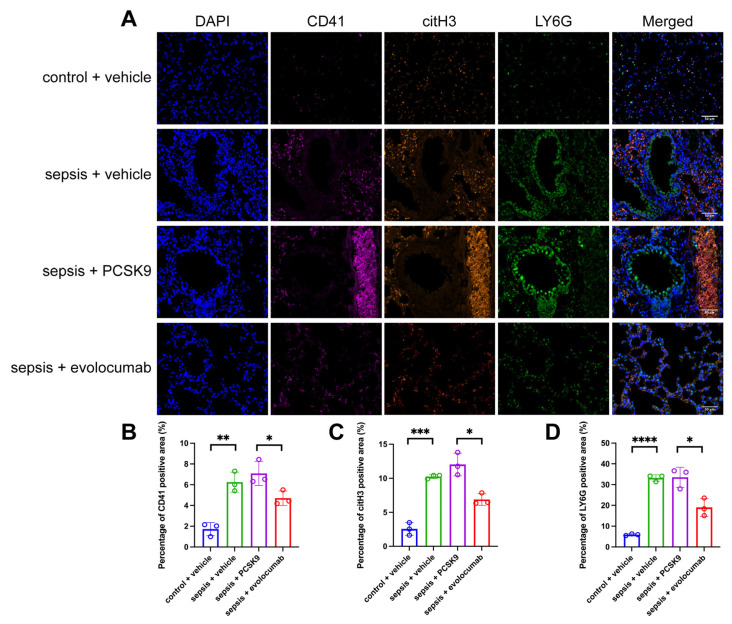
In the sepsis mice, neutrophil infiltration occurred in the lumen of pulmonary microvessels and around the vessel walls, NETs were formed, and platelets were aggregated. (**A**) Representative images of immunofluorescence staining of DAPI (blue), CD41 (magenta), citH3 (orange), LY6G (green) and colocalization in the lung tissues of the four groups, scale bar = 50 μm. (**B**) Quantification of the percentage of CD41-positive area among the four groups. (**C**) Quantification of the percentage of citH3-positive area among the four groups. (**D**) Quantification of the percentage of LY6G-positive area among the four groups. Data are shown as the mean ± SD (*n* = 3 mice/group). * *p* < 0.05, ** *p* < 0.01, *** *p* < 0.001, **** *p* < 0.0001 vs. the indicated groups.

In addition, in the sepsis + vehicle group, compared to the control + vehicle group, the percentage of CD41-positive area increased significantly, demonstrating a large aggregation of platelets (Figure 6B); the percentage of citH3-positive area increased significantly, indicating an increase in NETosis (Figure 6C). At the same time, the percentage of LY6G-positive area also increased significantly, indicating an increase in neutrophil infiltration (Figure 6D). The alveolar structure was severely damaged, with significant thickening of the alveolar walls and dilation and congestion of the blood vessels. Extensive alveolar and interstitial edema was present, along with widespread inflammatory cell infiltration. Exfoliation of bronchial epithelial cells and neutrophil infiltration within the lumen were observed. Pulmonary arterioles exhibited edema, epithelial cell shedding, fibrinoid necrosis of the vessel walls, and a large number of platelet aggregations. A similar situation can be observed in sepsis + PCSK9 group. However, PCSK9 inhibitor evolocumab can effectively ameliorate these conditions (Figure 6).

## 4. Discussion

In this study, we provided compelling evidence that PCSK9 exacerbated pulmonary microthrombosis in patients with sepsis by promoting platelet activation and subsequent formation of NETs. By establishing a complete mouse model of CLP, we found that the expression of PCSK9 and platelet activation were significantly increased during sepsis. It is worth noting that recombinant PCSK9 further enhanced these changes, while evolocumab’s inhibition of PCSK9 effectively reversed these changes, alleviating microthrombosis and lung inflammation. These findings highlight the key mechanism link between PCSK9 activity, platelet-neutrophil interaction and thrombotic inflammatory injury in sepsis.

In 2016, sepsis was redefined as life-threatening organ dysfunction resulting from a dysregulated host response to infection [28]. Despite the progress made in diagnosis and treatment, the high mortality rate in intensive care units remains a challenge. The activation of coagulation and inflammation are crucial physiological responses for host defense during sepsis [29]. Nearly all patients with sepsis exhibit coagulation abnormalities [30]. Sepsis-associated coagulopathies encompass a spectrum, ranging from subtle activation of coagulation, detectable only through sensitive markers of coagulation factor activation, to more pronounced coagulation disturbances. The latter may be evidenced by a modest decrease in platelet count and subclinical prolongation of global clotting times. The lungs are the primary and most frequently affected organs in sepsis, with approximately 50% of septic patients developing lung injury [31,32].

Among the potential mechanisms underlying tissue injury during the acute inflammatory response, substantial evidence supports a role for microvascular obstruction caused by thrombosis. Inflammation and thrombosis are closely interconnected processes [33]. Inflammatory activation enhances procoagulant pathways by upregulating factors such as tissue factor (TF), platelet reactivity, and fibrinogen levels. Concurrently, it downregulates natural anticoagulant systems, including protein C, protein S, thrombomodulin, and antithrombin, while inhibiting fibrinolysis through increased expression of plasminogen activator inhibitor-1 (PAI-1) [34,35]. Numerous clinical conditions exemplify this interplay between inflammation and thrombosis. Elevated C-reactive protein levels in patients with atherosclerotic disease are associated with a heightened risk of thrombosis-related cardiovascular events [35,36]. Similarly, acute transplant rejection frequently leads to thrombosis within the transplanted organ, and inflammation associated with sepsis can trigger disseminated intravascular coagulation (DIC) [29,37].

In addition to pathogen-induced activation of coagulation, several other critical pathways contribute to the pathogenesis of sepsis-induced pulmonary microthrombosis. These include the involvement of damage-associated molecular patterns (DAMPs), NETs, extracellular vesicles, and damage to the endothelial glycocalyx [2]. Our study showed that pulmonary alveolar inflammatory infiltration and fibrinoid necrosis of blood vessel wall were observed in mice with sepsis. However, the exact mechanism underlying these phenomena remains unclear.

Platelets are fragments derived from megakaryocytes that lack nuclei and are primed to maintain primary hemostasis by initiating blood clot formation on injured vascular endothelia. In addition to playing a central role in normal haemostasis, platelets play a crucial role in mediating the innate immune response to infection, a process referred to as immunothrombosis, which constitutes a normal physiological reaction to infection [29]. Thrombus formation is a multifaceted process that involves not only platelets but also cells of the innate immune system. The interplay between the innate immune response and thrombus formation is termed thrombo-inflammation [9]. We found that platelets in septic mice were in an activated state. Yet, the underlying cause of platelet activation remains unclear.

PCSK9, a serine protease predominantly synthesized in the liver, plays a pivotal role in lipid metabolism by degrading the low-density lipoprotein receptor (LDLR), thereby increasing plasma levels of low-density lipoprotein cholesterol (LDL-C) [38]. In addition to its role in LDL metabolism, several studies have highlighted the involvement of PCSK9 in various stages of atherosclerosis, particularly through its ability to target other members of the LDL receptor (LDLR) family. PCSK9 derived from both plasma and vascular cells contributes to the development of atherosclerotic plaques and thrombosis by promoting platelet activation, leukocyte recruitment, and clot formation, independent of systemic lipid changes. These findings further underscore the potential cardiovascular benefits of therapies aimed at inhibiting PCSK9 [39]. Our findings indicated that the expression of PCSK9 was increased in septic mice. Furthermore, administration of PCSK9 promoted platelet activation in these septic mice.

NETs are networks of histone-modified nuclear material released from activated neutrophils during inflammatory responses. These degranulation events can be directly induced by the interaction between platelets and neutrophils [40]. NETs were initially recognized as an overlooked defense mechanism of neutrophils, owing to their ability to entrap and potentially eliminate a broad range of pathogens. However, accumulating evidence now indicates that NETs are involved in various pathophysiological conditions, including autoimmunity, cancer, diabetes mellitus, and Alzheimer’s disease. Notably, NETs are also thought to play a critical role in the pathogenesis of atherosclerosis and thrombosis [41]. NETs closely interact with fibrin fibers within the thrombus, thereby influencing its organization and stability. Moreover, histones contained in NETs or released following NET-DNA degradation can stimulate platelet aggregation and activate the coagulation cascade [42,43]. The molecular mechanisms underlying NET formation remain inadequately understood; however, recent studies have demonstrated that platelets activated by lipopolysaccharide (LPS) can induce the formation of NETs [23,44]. Our study shows that platelet activation in sepsis mice promoted the production of NETs, which led to the development of pulmonary microthrombosis.

Study has shown that PCSK9 inhibition can reduce the mortality rate of septic mice by eliminating endotoxins [45]. Qi Z et al. found that PCSK9 level has a certain effect on promoting platelet activation in sepsis [25]. In addition, Existing research has discovered that PCSK9 deficiency has been associated with a reduced risk of venous thrombosis, potentially mediated through decreased leukocyte recruitment and attenuated formation of NETs at the site of thrombus development [46]. Our study demonstrated that administration of PCSK9 led to platelet activation, which subsequently promoted the formation of NETs, thereby inducing thrombosis and ultimately resulting in pulmonary microthrombosis in septic mice. Notably, the administration of the PCSK9 inhibitor evolocumab effectively alleviated the pathological changes caused by sepsis and inhibited the onset and progression of pulmonary microthrombosis. This study provides new insights into the role of PCSK9 in regulating platelet activation and NET formation and offers novel findings regarding the involvement of PCSK9 in sepsis-induced pulmonary microthrombosis. These data may enhance our understanding of pulmonary microthrombosis associated with sepsis.

Our data show that the expression of PCSK9 in septic mice is significantly elevated, which is consistent with the previously reported elevated levels of circulating PCSK9 in patients with sepsis or systemic inflammation [15,26]. The upregulation of PCSK9 is associated with a hypercoagulable state, as evidenced by TEG parameters (reduced R-time and k-time, increased α-angle and MA values). These data support the hypothesis that PCSK9 promotes thrombosis during sepsis. ELISA detected an increase in PF4 levels, further confirming platelet activation. This suggests that PCSK9 not only regulates lipid metabolism but also directly promotes platelet procoagulant activity.

In terms of mechanism, multiple studies have shown that PCSK9 can interact with the platelet surface receptor CD36, triggering oxidative stress and the activation of downstream p38MAPK and JNK pathways, thereby enhancing platelet aggregation [25,47]. Our research results are consistent with this mechanism—in the TEG trial, the administration of recombinant PCSK9 led to an increase in PF4 release and enhanced clot structure, which implies enhanced platelet reactivity. On the contrary, evolocumab significantly normalized these parameters, indicating that inhibiting PCSK9 can alleviate platelet hyperactivity in patients with sepsis.

In addition to platelet activation, our immunofluorescence and ELISA analyses confirmed a significant increase in NET formation, as indicated by elevated levels of MPO and citH3. This observation is consistent with previous studies [20,23], indicating that activated platelets can induce NETs release through TLR4-mediated signal transduction. The co-localization of CD41 (platelet marker) and citH3 (NETs marker) in pulmonary microvessels supports the synergistic effect of platelets and neutrophils in microthrombosis. Importantly, evolocumab treatment significantly reduced NETs accumulation, reinforcing the concept that PCSK9 inhibition blocked the platelet-neutrophil axis and suppressed thrombophilia.

Histopathological analysis further indicated that PCSK9 exacerbated the thickening of alveolar walls, vascular congestion and fibrinoid necrosis in septic lung, all of which are hallmark features of microthrombotic injury. These pathological improvements after evolocumab administration indicate that PCSK9 activity not only affects intravascular coagulation but also promotes secondary tissue damage through inflammation and endothelial pathways. Previous studies have shown that PCSK9 enhances TLR4/MyD88/NF-κB signaling and endothelial dysfunction during sepsis [26,48]. Our data expand this concept by linking PCSK9-mediated inflammation to platelet NETs-driven microvascular thrombosis.

In summary, our research results propose a working model by which PCSK9 amplifies sepsis-induced lung injury through a dual mechanism: (1) Directly activating platelets and enhancing coagulant and adhesion properties; (2) Promote the formation of NETs, and facilitate microvascular occlusion and endothelial injury. Inhibition of PCSK9 disrupts this pathological cycle, highlighting its potential as a therapeutic target for septic microthrombosis.

Our experimental data provide strong support for our conclusion. However, further research on the use of viral vector-mediated PCSK9 overexpression in immature mice to assess whether these phenotypic changes alone cause thrombosis may provide additional evidence to strengthen our findings. It is important to be aware of the limitations of our research. Although our in vivo data clearly demonstrated the contribution of PCSK9 to platelet activation and NET formation, this study did not fully distinguish whether PCSK9 acts directly on neutrophils or indirectly on platelet-derived mediators. Furthermore, we did not assess the circulating cytokine levels of platelets and neutrophils or specific intracellular signaling pathways. Future research will require the use of cell type-specific PCSK9 knockout models or in vitro co-culture systems of platelets and neutrophils to directly investigate the pathogenic role of PCSK9 in NET formation and describe the precise molecular mechanisms involved.

## 5. Conclusions

In conclusion, our research results indicate that PCSK9 is a key amplifier of platelet activation and NET formation during sepsis, thereby exacerbating pulmonary microthrombosis. The pharmacological inhibition of PCSK9 by evolocumab effectively reversed these effects, providing a promising therapeutic strategy for alleviating thrombotic complications in sepsis-induced lung injury. Targeting the PCSK9–platelet–NETs axis may offer a novel therapeutic approach for the prevention and treatment of sepsis-Induced lung Injury and thrombotic complications.

## Data Availability

Data supporting the findin.gs of this study are available within the article.
